# Well-Differentiated Liposarcoma of the Psoas Muscle: A Distinct Anatomic Subset with Highly Favorable Outcomes

**DOI:** 10.1245/s10434-026-19738-3

**Published:** 2026-05-05

**Authors:** William W. Tseng, Francesco Barretta, Marco Fiore, Stefano Radaelli, Marco Baia, Alessandra Borghi, Chiara Colombo, Roberta Sanfilippo, Chiara Fabbroni, Silvia Stacchiotti, Elena Di Blasi, Claudia Sangalli, Albina Allajbej, Carlo Morosi, Andrea Vanzulli, Sandro Pasquali, Dario Callegaro, Alessandro Gronchi

**Affiliations:** 1 Division of Surgical Oncology, Department of Surgery, City of Hope National Medical Center, Duarte, CA USA; 2 Unit of Biostatistics for Clinical Research, Fondazione IRCCS Istituto Nazionale dei Tumori, Milan, Italy; 3 Sarcoma Service, Department of Surgery, Fondazione IRCCS Istituto Nazionale dei Tumori, Milan, Italy; 4 Department of Medical Oncology, Fondazione IRCCS Istituto Nazionale dei Tumori, Milan, Italy; 5 Medical Sarcoma Unit, IRCCS Istituto Europeo di Oncologia, Milan, Italy; 6 Division of Pathology, IRCCS Istituto Europeo di Oncologia, Milan, Italy; 7 Department of Radiation Oncology, Fondazione IRCCS Istituto Nazionale dei Tumori, Milan, Italy; 8 Department of Radiology, Fondazione IRCCS Istituto Nazionale dei Tumori, Milan, Italy; 9 Molecular Pharmacology, Department of Experimental Oncology, Fondazione IRCCS Istituto Nazionale dei Tumori, Milano, Italy

**Keywords:** Liposarcoma, Well- Differentiated, Psoas, Retroperitoneal Sarcoma

## Abstract

**Background:**

Retroperitoneal (RP) liposarcoma (LPS) is characterized by histologic subtype and grade. This study sought to define outcomes for a novel subset of well-differentiated (WD) RP LPS defined by anatomic location in the psoas muscle.

**Methods:**

Data were reviewed for all patients with primary WD LPS who underwent curative-intent complete resection at our sarcoma referral center from March 2006 to September 2021. For the study patients, clinicopathologic data were summarized. Crude cumulative incidences of local recurrence (LR) and disease-specific death (DSD) were estimated.

**Results:**

The 254 patients who met the inclusion criteria were stratified by location of their tumor: 12 RP psoas patients (group A), 123 RP non-psoas patients (group B), and 119 extremity patients (group C). Tumor size was greater in groups A (median, 20.0 cm) and B (median, 25.0 cm) versus group C (median, 15.0 cm; standardized mean difference [SMD], 0.747). Four patients (33 %) in group A underwent concomitant resection of muscle or nerve. In group B, 120 patients (97.5 %) had at least one organ resected (SMD, 5.606). During a median follow-up period of 8.8 years, no LR occurred in group A. The 5-year incidence of LR was 16.3 % in group B and 2.9 % in group C (*p* < 0.001). No DSDs occurred in group A or C, whereas the 5-year incidence of DSD was 1.9 % in group B.

**Conclusions:**

In psoas WD LPS, local control rates and survival are highly favorable after surgery and approximate those seen for patients with extremity disease. With further multicenter validation, a more conservative approach to management may be indicated for this anatomically defined subset of RP liposarcoma.

Retroperitoneal (RP) liposarcoma (LPS) is a rare adipocytic malignancy that occurs in the back of the abdomen. Surgery is the mainstay of treatment, but the overall incidence of local recurrence is high, reaching 50–60 % at 5 years.^1,2^ At specialist centers, wider resection that includes adjacent organs (e.g., ipsilateral kidney, colon) when feasible and safe can optimize the “soft tissue margins” surrounding the tumor to improve local control.^3,4^ Preoperative radiation therapy also can be used, and in a recent prospective multicenter trial (STRASS), this approach was shown to improve local control compared with surgery alone for select patients with RP LPS.^5^

The approach to treatment, however, is not uniform and must account for heterogeneity within RP LPS that significantly impacts clinical outcome.^6^ RP LPS is characterized histologically, distinguishing between well-differentiated (WD) and dedifferentiated (DD) liposarcoma by WHO classification, and Evans criteria. The Fédération Nationale des Centres de Lutte Contre le Cancer (FNCLCC) system is widely applied for histological grading of DD liposarcomas, based on tumor differentiation, mitotic count and necrosis. Dedifferentiated liposarcoma is most commonly associated with a well differentiated component; notably, even in tumors that are predominantly well differentiated, the presence of a DD component changes the disease biology such that the incidence of local recurrence is markedly higher, and the potential for distant metastasis is conferred in contrast to tumors that are entirely WD. In addition, histomorphologic variants also are recognized in WDLPS, with differential clinical outcomes; the sclerosing variant of WD LPS is associated with a worse outcome, whereas the lipoma-like variant of WD is associated with a better outcome.^7,8^

In this study we sought to determine whether anatomic location within the RP can be used to characterize a subgroup of LPS with a distinct clinical outcome. To our knowledge this has not been done previously. We focused our attention on the relationship of the tumor to the psoas muscle, a muscle in the back of the abdomen, adjacent to the spine and involved in hip flexion.

For the purposes of this study, only patients with WD disease in the initial primary disease setting were included. This was done in part to facilitate comparison with LPS in the extremity. Compared with the RP location, WD/DD LPS in the extremity is well-recognized to have a better outcome.^9–12^ The frequency of WD in the extremity is much higher than that of DD, and as such can be a useful group for comparison with RP psoas WD LPS.

## Methods

### Patient Selection

Under institutional review board approval, a prospectively maintained database of soft tissue sarcoma patients was used to retrospectively collect data on patients with LPS who underwent surgery at the Fondazione IRCCS Istituto Nazionale dei Tumori (INT) in Milan, Italy, a high-volume sarcoma referral center, from March 2006 to September 2021. The diagnosis of LPS was made based on histopathologic examination coupled with positive MDM2 expression by immunohistochemistry or amplification of this gene by fluorescence *in situ* hybridization. High-level MDM2 amplification is pathognomonic for WD/DD LPS and rules out other rarer subtypes (e.g., myxoid round cell or pleomorphic LPS).

**Fig. 1:**
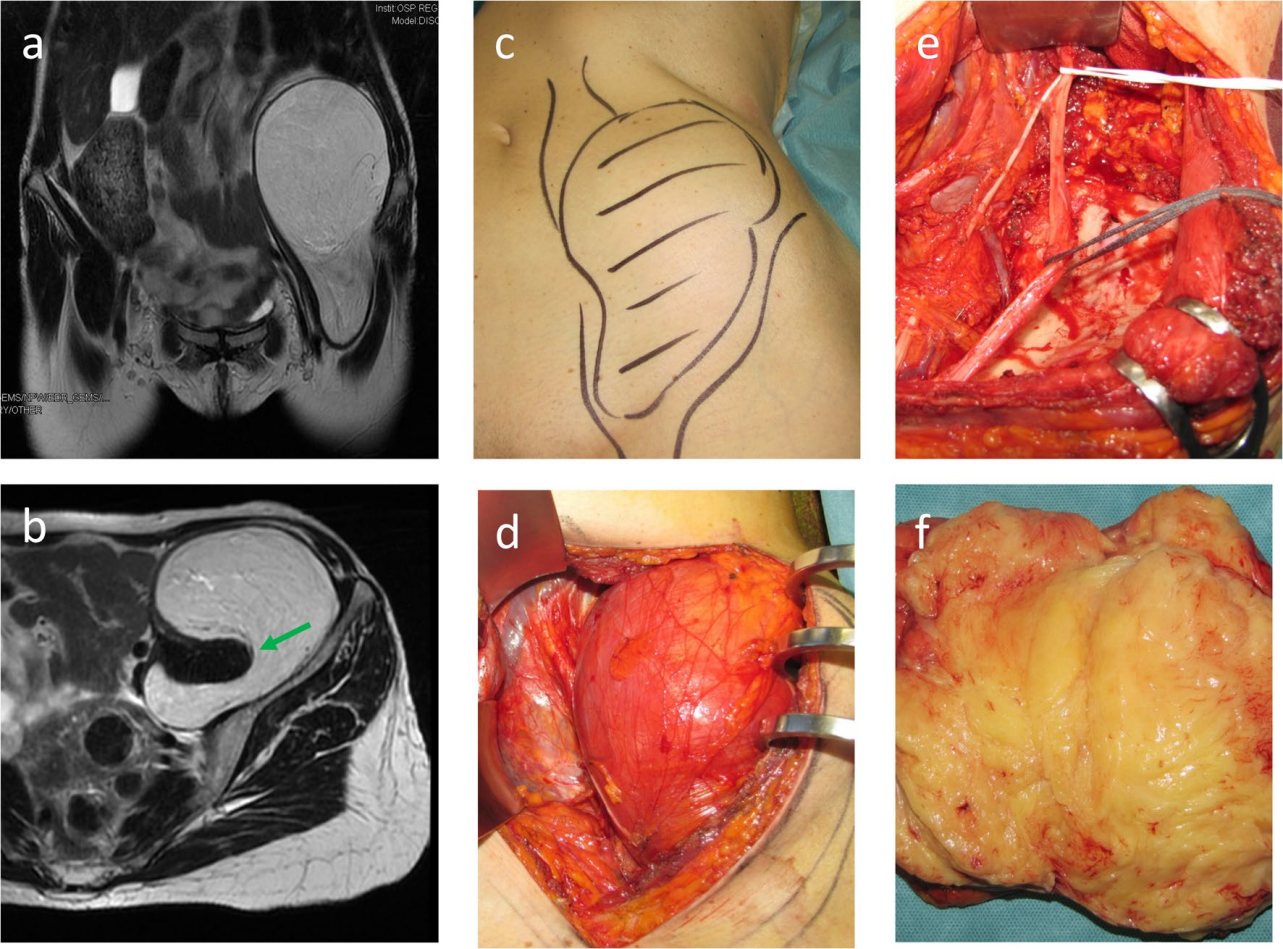
Representative case of well-differentiated liposarcoma of the psoas. Coronal and axial MRI images (**a, b**) showing the tumor in relation to the left psoas muscle (green *arrow*). Intraoperative photos (**c, d**) and operative field after tumor resection (**e**) with preservation of the femoral nerve. Gross tumor sectioned to demonstrate an entirely lipomatous mass (**f**) that on pathology demonstrated histologic features and amplification of MDM2 (not shown) supporting the diagnosis

From this pathology-verified dataset we included only primary WD disease and patients who underwent curative-intent, complete resection (R0/R1). The study excluded patients with initial presentation of DD, those with recurrent disease, and those who underwent palliative-intent, piecemeal or incomplete resection (R2) at the time of surgery at the INT. We then focused on the anatomic location to include the retroperitoneum and intraabdominal/pelvic space versus the extremity, excluding all other sites (e.g., trunk, head and neck, mediastinal). After careful re-review of the preoperative imaging and intraoperative findings, we also identified a subset of patients with RP tumors confined to or arising in the ipsilateral psoas muscle (example shown in Fig. 1).

### Statistical Analysis

The primary study endpoints were crude cumulative incidence of local recurrence (CCI LR) and disease-specific death (CCI DSD) and overall survival (OS).

The time of risk for LR and DSD began at the date of surgery. Distant metastasis and death from any cause was considered as a competing event for LR. Concomitant LR and DM were considered as DM. Death from other cause was considered as a competing event for DSD. The study defined DSD as death from either local or distant disease as well as death due to complications. Death of a patient from an unknown cause was classified as death from other causes. Time was censored at the date of the last follow-up visit for patients remaining alive and free of any event. Crude cumulative incidence curves were estimated in a competing risk setting and compared using the Gray test.

Overall survival (OS) was defined as the time between surgery and death from any cause. Time was censored at the date of the last follow-up visit for patients remaining alive. Overall survival curves were estimated using the Kaplan-Meier method and compared using the log-rank test.

Kaplan-Meier and CCI curves were compared according tumor site. Estimates of OS and CCI were reported as percentages with 95 % confidence intervals (CIs).

Patient and disease characteristics and treatments were summarized by descriptive statistic: overall and by tumor site (RP psoas, RP non-psoas, extremity). Categorical variables were summarized using absolute and relative frequencies. Continuous variables were summarized using the median and the first and third quartiles, commonly denoted as the interquartile range (IQR). The standardized mean difference (SMD) was used as a measure of between-group differences.^13^ The SMD was considered to indicate a possible between-group imbalance at a value of about 0.3. For SMD values of 0.3 or higher, it was important to assess the clinical or practical importance of such differences.

Median follow-up times were estimated by the reverse Kaplan-Meier method from the OS data.^14^ The statistical analyses were performed with SAS (v. 9.4; SAS Institute, Cary, NC, USA) and R software (v. 4.5.1; R Foundation for Statistical Computing, Vienna, Austria).

## Results

### Patient and Tumor Characteristics

During the study period (2006–2021), 371 patients with primary WD LPS underwent curative-intent surgery at our center. After applying exclusion criteria, our final study cohort included a total of 254 patients with WD LPS of the RP or extremity. The patients with RP WD LPS were further stratified by location of their tumor in relation to the psoas muscle, resulting in three groups: group A (RP psoas: *n* = 12, 4.7 %), group B (RP non-psoas; *n* = 123, 48.4 %), and group C (extremity: *n* = 119, 46.9 %).

Demographic and clinicopathologic data for the study patients are summarized (Table 1). The RP patients (groups A and B) and the extremity patients (group C) were similar in age (median age, respectively 62 and 61 years vs 59 years; SMD, 0.018), whereas tumor size was larger in groups A and B (median psoas [20.0 cm] and non-psoas [25.0 cm] vs group C (median extremity [15.0 cm]; SMD, 0.747).

**Table 1:** Demographics and clinical variables

	All	RP psoas	RP non-psoas	Extremity	SMD
	(*n* = 254)	(*n* = 12)	(*n* = 123)	(*n* = 119)	
	*n* (%)	*n* (%)	*n* (%)	*n* (%)	
*Median age: years*					0.018
(IQR)	60 (52.0–68.0)	62 (52.8–68.8)	61 (52.0–69.0)	59 (52.0–68.0)	
Sex					0.085
Female	120 (47.2)	5 (41.7)	59 (48.0)	56 (47.1)	
Male	134 (52.8)	7 (58.3)	64 (52.0)	63 (52.9)	
*Median tumor size: cm*					0.747
(IQR)	20 (14.0–27.0)	20 (15.5–21.0)	25 (20.0–30.0)	15 (11.0–20.5)	
*Organs resected*					5.606
0	130 (51.2)	8 (66.7)	3 (2.4)	119 (100.0)	
≥1	124 (48.8)	4 (33.3)	120 (97.5)	0 (0.0)	
*Clavien-Dindo score*					0.708
<3	211 (83.1)	12.0 (100.0)	81 (65.9)	118 (99.2)	
≥3	43 (16.9)	0 (0.0)	42 (34.1)	1 (0.8)	

RP, Retroperitoneal; SMD, Standardized mean difference; IQR, Interquartile range

All the patients underwent complete R0/R1 resection of their tumor. The surgery for the 12 group A patients included concomitant resection of muscle in three patients and femoral nerve in one patient (33 %, 4/12). For the remaining eight patients (67 %), tumor resection alone was performed. In contrast, in group B, concomitant resection of at least one visceral organ or structure was performed in 120 (97.5 %) of 123 patients (SMD, 5.606). No patients in group A versus 42 patients (34.1 %) in group B experienced major postoperative complications (Clavien-Dindo grade ≥3).

### Outcomes

The median follow-up period was 8.8 years for group A (RP psoas), 8.7 years for group B (RP non-psoas), and 6.7 years for group C (extremity). During the study period, LR occurred for 0 of 12 patients in group A (0.0 %), 38 of 123 patients in group B (30.9 %), and 7 of 119 patients in group C (5.9 %). Disease-specific death occurred for 0 patients in groups A and C versus 9 patients in group B (7.3 %). Death due to any cause occurred for 1 patient in group A (8.3 %), 20 patients in group B (16.3 %), and 2 patients in C (1.7 %). The one patient in group A died of pancreatic adenocarcinoma, unrelated to the RP psoas WD LPS.

**Fig. 2:**
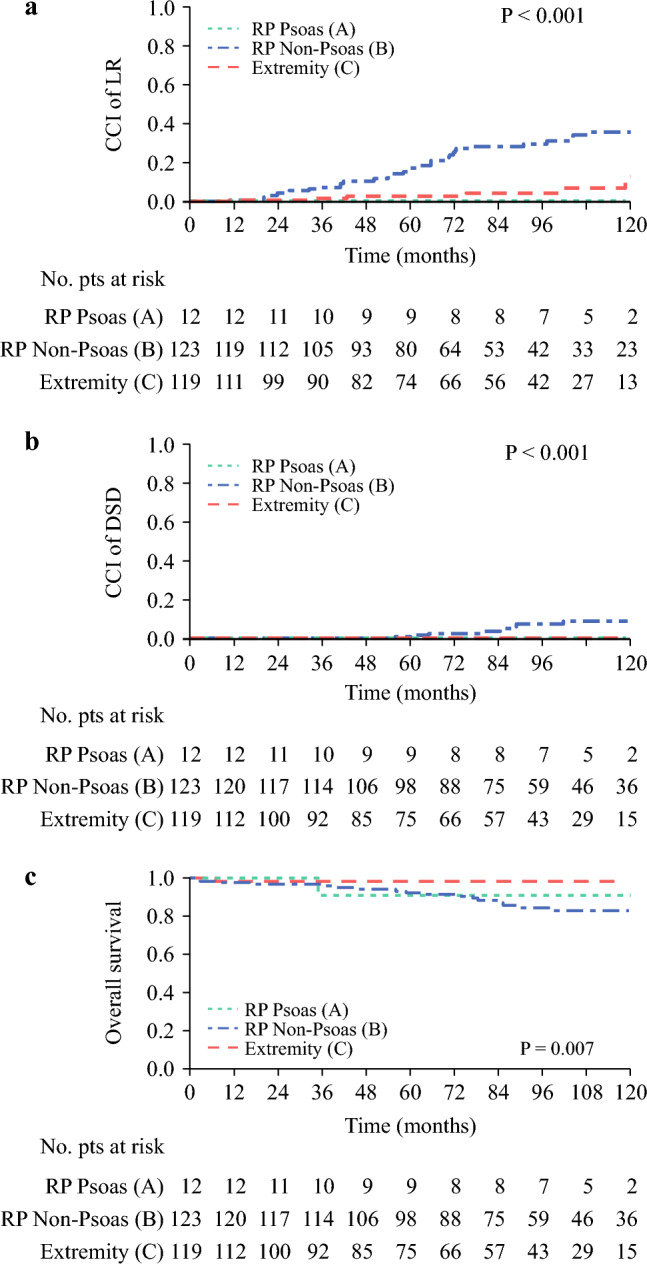
Clinical outcome of the study patients stratified by group based on anatomic location of their tumor. Crude cumulative incidence curves of local recurrence (**a**) and disease-specific death (**b**), and Kaplan-Meier curves of overall survival (**c**)

Crude cumulative incidences of LR and DSD are shown in Fig. 2a and b. The 5-year incidence of LR for the RP patients was 0.0 % in group A versus 16.3 % in group B and 2.9 % in group C (*p* < 0.001; Fig. 2a). The 5-year incidence of DSD was 0.0 % in groups A and C versus 1.9 % in group B (*p* < 0.001; Fig. 2b). The OS for each group is shown in Fig. 2c (*p* = 0.007).

## Discussion

The current study suggests that in addition to standard characterization of RP LPS by histologic subtype and grade, the specific anatomic location of the tumor (in this case, the relationship to the psoas muscle) has potential implications for clinical outcome. With surgery as the mainstay of treatment, patients with psoas WD LPS appear to do extremely well with no local recurrence or DSD during a median follow-up period of almost 9 years. The disease is essentially cured, a goal that is otherwise highly elusive for the majority of patients with RP LPS.^6^ As highlighted by the lower frequency of concomitant resection of organs and adjacent structures (33.0 % vs. 97.5 %), the extent of surgery in psoas the WD LPS cases was in fact rather conservative. Moreover, on post hoc re-review, none of the patients with psoas WD LPS received radiation or chemotherapy.

Our study used data from a single high-volume sarcoma referral center, which permitted accumulation of enough patients with this rare subset of RP LPS (12 of 371, 3.2 % of all WD LPS, any location) and controlled for a standardized management approach, including access to multidisciplinary expertise. However, a critical limitation was the retrospective nature of the data and potential inherent biases. The study period of 15 years spanned adoption of extended or compartmental resection (e.g., systematic resection of adjacent organs/structures even if not overtly involved) and the STRASS trial (neoadjuvant radiation therapy).^3,5^ None of the psoas WD LPS patients underwent extended resection or were enrolled in STRASS, implying that there may have been upfront bias in the treatment approach from the operating surgeon. In addition, the surgical approach in most of these psoas WD LPS cases was lateral (extraperitoneal) versus through a midline laparotomy.

**Fig. 3:**
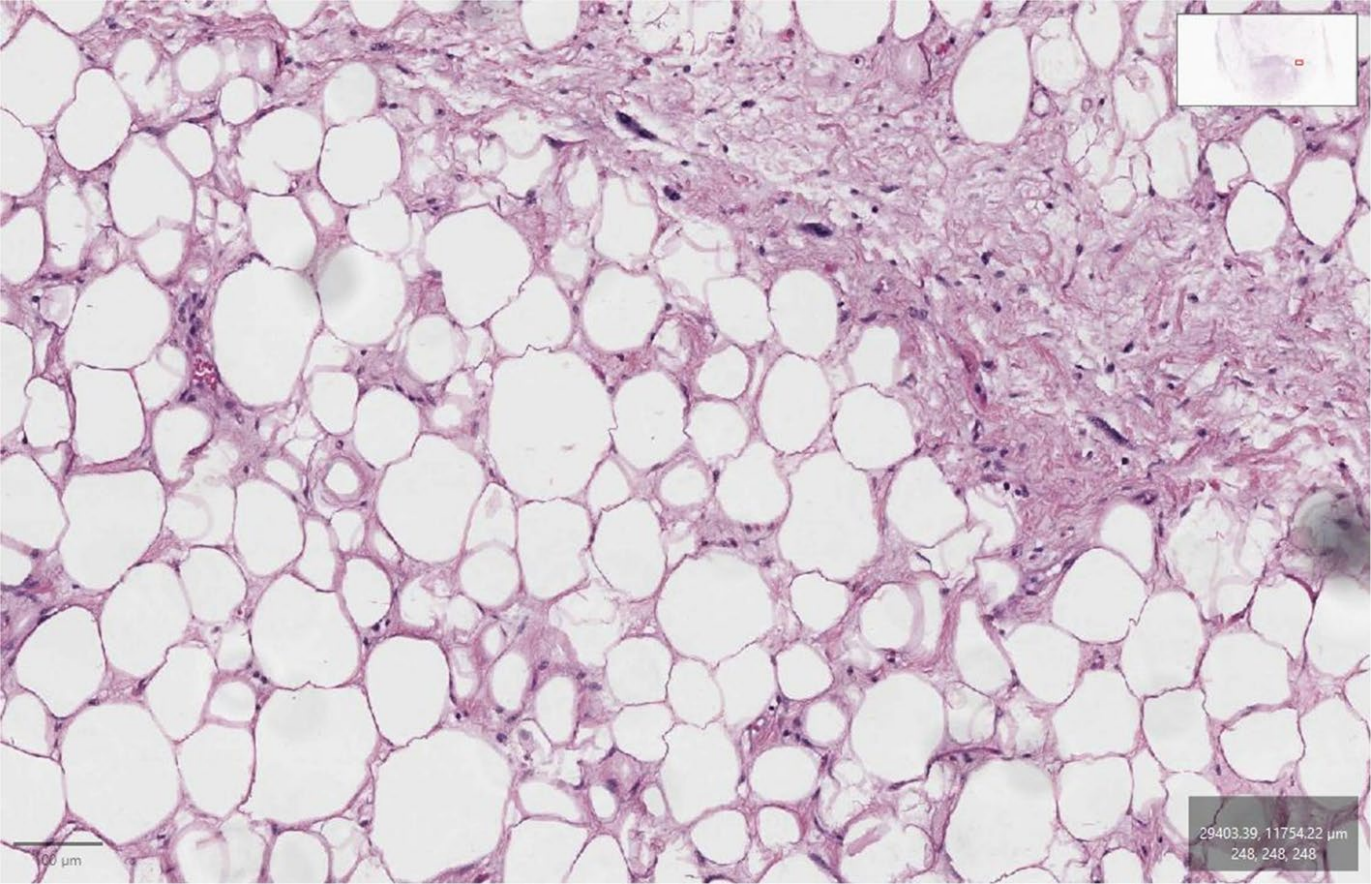
Representative histomorphology for one patient with well-differentiated liposarcoma of the psoas. Concurrent lipoma-like (*lower left*) and sclerosing areas (*upper right*) are demonstrated

Clinical outcomes in RP LPS may be impacted by histomorphologic variance.^7,8^ In an earlier study of extremity and truncal WD LPS, lipoma-like versus sclerosing tumors were associated with a 100 % versus a 78 % 5-year LR-free survival (*p* = 0.01).^7^ In the current study, however, the extent of this impact for the psoas WD LPS group was unclear. Post hoc re-review of the pathology of the 12 cases demonstrated seemingly discordant overlap: 8 (66.7 %) had concurrent lipoma-like and sclerosing features (Fig. 3). Four cases also had a prominent inflammatory component. One case had osseous and cartilaginous differentiation. Further substratification (e.g., by extent of sclerosis^8^) and deeper molecular investigation is ongoing.

Overall, an intriguing observation from our data was that the outcome for patients with psoas WD LPS approximated that seen for extremity WD LPS patients. In fact, this approximation (e.g., incidence of LR: 0.0 % vs 2.9 % at 5 years) appeared to be stronger than that observed for “conventional” (non-psoas) RP LPS (LR: 16.3 % at 5 years). This raises the question whether psoas WD LPS, despite the RP location, should be regarded and managed similarly to extremity disease, which can also be confined to muscle (e.g., quadriceps, hamstrings) or arise from muscle. The current standard approach for these “atypical lipomatous tumors (ALT)” as they are often referred to by orthopedic oncologists who treat these tumors, is in fact conservative.^15^ Although none of the 12 patients with psoas WD LPS in our study received radiation therapy, some reported evidence exists to suggest that this may be considered to further optimize local control in extremity ALT.^16^

In conclusion, based on the current study findings, the heterogeneity of RP LPS is further nuanced by anatomic location in the psoas muscle for WD tumors. With further multicenter validation, a more conservative approach to the management of patients with psoas WD LPS may be warranted, including resection of adjacent organs or structures only if obviously inseparable and omission of neoadjuvant or adjuvant therapies if complete (R0/R1) resection is anticipated or achieved. Whether the term “psoas ALT” should be adopted remains debatable, but certainly recognition of this distinct subset with highly favorable outcomes should be made in studies of RP LPS moving forward. Finally, from a translational standpoint, further research is warranted to explore differences between these anatomically defined tumors and WD tumors in general and benign large lipomas. There may be mechanistic reasons (e.g., level of MDM2 amplification or another novel biomarker) that underlie the differences in clinical outcome.
